# Circular economy through waste reverse logistics under extended producer responsibility in Finland

**DOI:** 10.1177/0734242X231168801

**Published:** 2023-04-29

**Authors:** Bening Mayanti, Petri Helo

**Affiliations:** 1 Vaasa Energy Business Innovation Centre, University of Vaasa, Vaasa, Ostrobothnia, Finland; 2 Department of Production, University of Vaasa, Vaasa, Ostrobothnia, Finland

**Keywords:** Waste reverse logistics, extended producer responsibility, circular economy, waste management, policy instruments

## Abstract

Extended producer responsibility (EPR) is commonly implemented as a strategy in waste management. The core of the concept itself is a waste reverse logistics (WRL), which dictates how the collection, inspection and processing of end-of-life products are performed. Existing studies of EPR mainly focused on single products instead of using broader perspective on national level. Its contribution towards circular economy through slowing and closing the loops also has not been widely discussed. This study examined the system architecture of the policy instruments used in the EPR and the similarities of the WRL networks across different products. A case study was used to investigate six products: portable batteries and accumulators, paper, packaging, vehicles, electrical and electronic equipment (EEE) and tyres. The study generated a WRL framework. It is also observed that closing the loop through recycling is the primary circular strategy and is found in all products, whereas closing and slowing the loop strategy through reuse/repair, remanufacture and repurposing is found in packaging, tyres, vehicles and EEE. This study shows that EPR can close the material loop, although improvement in design for the environment is necessary. It creates challenges and opportunities for the government, producer responsibility organization and producers to improve existing conditions by implementing new initiatives such as design for the environment indicators, standardization, tax and subsidy systems and tariffs for disposal fees.

## Introduction

Currently, the circular economy (CE) is seen as a solution to delink economic growth and environmental pressure (Hofmann, 2019). Previous studies on 114 definitions showed that the concept is widely perceived as end-of-life (EoL) management with strong focus on the recycling practice (Kirchherr et al., 2017). In contrast, some argued that the core of CE concerns resource management and value retention (Morseletto, 2020; Vermeulen et al., 2018). It can be implied that EoL management is a part of CE, and its value retention principle covers product life cycle throughout value chain. Nevertheless, the strong conception that identify CE as EoL is reasonable considering the state of global waste management (WM) where approximately two-thirds of the waste still go into landfill and open dumping (The World Bank, 2018), putting WM on the spotlight under the realm of CE.

Extended producer responsibility (EPR) is an environmental principle that requires policy instruments for its application to achieve sustainable WM (Lindhqvist, 2000). It started as a mechanism to shift responsibility in handling waste from municipalities to the producers, progressing to the present day as a means to advance collection and its subsequent treatment as well as design for the environment (DfE) and closing the loop (Lindhqvist, 2000; Massarutto, 2007; The Organization for Economic Cooperation and Development (OECD, 2016)). EPR in Europe includes a directive for electrical and electronic equipment (EEE), vehicles, batteries and accumulators and packaging (Deloitte, 2014). It is rooted in the European Union (EU) waste framework directive (Directive 2008/98/EC), which adopts different policy instruments in implementing the EPR.

EPR is critically important to facilitate funding that is dedicated to ensure proper collection, sorting and recovery of EoL products. Various studies have been done regarding EPR implementation such as tyres (Campbell-Johnston et al., 2020; Winternitz et al., 2019), packaging (Rubio et al., 2019), lamps (Richter and Koppejan, 2016), vehicles (Santini et al., 2011; Xiang and Ming, 2011) and e-waste (Leclerc and Badami, 2020; Wang et al., 2017). However, the majority of studies focused on EPR performance of a single product, and few of them studied a couple of products. As a result, there is a lack of perspective regarding challenges, barriers and performance of overall EPR implementation on a national level. Moreover, existing studies have not yet looked into the network and policy instruments. As far as the authors are aware, no study has been conducted to investigate policy instruments and the reverse logistics (RL) network of collective EPR in Finland. Hence, this study aims to examine the waste reverse logistics (WRL) networks of the collective EPR system within the Finnish context. It intends to contribute towards CE and provide knowledge to improve EPR implementation, such as setting new targets or applying new policy instruments. This goal is achieved by answering the following questions: (1) What is the system architecture of the policy instruments? and (2) How different or similar is the WRL network across different EoL products? Multiple analyses of the system architecture and WRL network are developed for six products under the EPR scheme: portable batteries and accumulators, paper, packaging, vehicles, EEE and tyres.

## Literature overview

### Waste reverse logistics

RL deals with the flow of products from consumers because the products are defective, unwanted, recalled or discarded after use (De Brito and Dekker, 2004). In the context of WM, it deals with discarded products. WM is mostly about effective and efficient activities to prevent, collect, transport, recover and treat waste, whereas RL is concerned with recapturing value and directing it back to the market (Seadon, 2010; Triantafyllou and Cherrett, 2010). Nonetheless, overlaps occur between WM and RL, as shown by definition evolving throughout the years. Within CE framing, WM and RL have contributions since CE emphasizes that waste is a resource and the remaining value should be captured and re-entered the supply chain (Potting et al., 2017).

RL comprises four main activities: gatekeeping, collection, sorting and recovery. Gatekeeping is a typical return management activity where the company screens the returned products to decide whether those products are allowed to enter the reverse supply chain (Hjort, 2010). In WRL, the activity starts with the collection since all products will enter the reverse channel once discarded, regardless of the condition. Collection refers to transferring products from the source to where further treatment is applied (Barker and Zabinsky, 2008). The collection scheme can be the curbside, drop-off or pick-up method. The collected products are sorted based on quality to determine the following recovery options. The last step is recovery, which can be done on different product structure levels, such as whole product, component and material. In CE, the recovery level is expected to maintain the use of the product and material as long as possible through reuse, repair, refurbishing, remanufacturing, repurposing, recycling and energy recovery (Potting et al., 2017).

### Extended producer responsibility

EPR is an extension of the ‘polluter pays principle’ whose implementation requires policy instruments to incentivize producers in preventing waste generation (Campbell-Johnston et al., 2020). It intends to internalize an externality of EoL costs where the price typically does not capture all the costs (OECD, 2016). Policy instruments required in EPR are varied, and they define EPR efficiency. Different types of policy instruments in EPR implementations are shown in Table 1 (OECD, 2016; Tojo, 2004).

**Table 1.** Examples of policy instruments to implement extended producer responsibility.

Policy instruments	Examples
Administrative	Prohibition, recycling targets, regulation, take-back systems
Economic	Deposit–refund system, disposal fee (e.g. collection fee or recycling fee), tax
Informative	Eco-labelling, research and development, reporting

Administrative instruments can be described as the command-and-control type that involves implementing particular tasks or banning certain activities (Tojo, 2004). Common examples include landfill bans, take-back schemes or recycling targets. Economic instruments offer a monetary incentive to implement certain tasks or financial disincentives to refrain from performing certain activities (OECD, 2016). Examples of economic instruments include taxing virgin material, paying an additional price when purchasing products, then receiving the payment back when the products are returned (deposit–refund system), or advanced disposal fees to finance waste treatment (Forslind, 2005; OECD, 2016). Informative instruments affect EPR indirectly through knowledge provision about the products and their impact on the environment (Lindhqvist, 2000). An example of an informative instrument is eco-labelling, which targets behavioural change when consumers better understand products and their related impact (Tojo, 2004).

A study by OECD (2016) showed that three-quarters of EPR implemented various forms of take-back systems that can be done individually or collectively. The individual scheme means that the producers take responsibility for the EoL management of their products; meanwhile, a collective scheme occurs when the producers of the same products assemble to manage the EoL of their products with the help of a third-party organizer called producer responsibility organization (PRO) (Tojo, 2003). Deposit–refund system and advanced disposal fee cover almost the rest. A mix of different policy instruments is also commonly used. For example, beverage packaging in Finland implements a combination of three policy instruments. It applies a take-back scheme that primarily utilizes return vending machines, a deposit–refund system to incentive product return, a target for reuse and recovery and reporting to authorities (Palpa, 2019).

EPR has played a leading role in improving WM performance, as shown by relatively high collection and recycling rates (Niza et al., 2014; Tencati et al., 2016). Nonetheless, the collection rate and processing rate (recycling or recovery) remain varied significantly across different products among EU members. Examples can be drawn from the recycling rate of packaging and vehicle in 2018, where the performance ranged between 36–85% and 72–91%, respectively (European Commission, 2021). Existing studies on EPR have focused on different aspects such as financing, evaluation of performance, challenges, a performance comparison between countries and responsibility fulfilment (Ahlers et al., 2021; Deloitte, 2014; Kunz et al., 2018). These studies mainly focused on one type of product, such as waste electrical and electronic equipment (WEEE) (Andersen et al., 2020; Cahill et al., 2011; Corsini et al., 2017; Rotter et al., 2011), tyres (Campbell-Johnston et al., 2020; Winternitz et al., 2019), packaging (Cahill et al., 2011; Rubio et al., 2019), end of life vehicle (ELV) (Forslind, 2005; Xiang and Ming, 2011) and lamps (Richter and Koppejan, 2016). Focusing on one product allows for deep analysis leading to more detailed results. However this study, which examined EPR on a country level, provided insights into the differences and similarities of each WRL network, including an evaluation to improve its performance.

### WRL network design under collective EPR

Policy instruments concerning EPR implementation influence the decision made by various actors. These actors include the government, producers, PRO, consumers, logistics and recycling operators (Forslind, 2005). Particular actors can impose instruments, while others can be affected directly by the instruments or indirectly through the actions taken by other actors. These decisions can affect how producers or PROs handle EoL products, shaping the WRL network (OECD, 2016).

The governments as actors can impose and enforce official order in the form of specific instruments or regulations that affect multiple actors. They can mandate producers to arrange a free-of-charge collection system which will affect the producers that have to bear the economic responsibility of collection and the subsequent treatment; hence, added fee is included at the point of sale (advanced disposal fee). This set of regulations from the government will be translated into strategies by the producers or PRO to meet EPR requirements and advocate for consumers to participate accordingly. The actions taken by producers and PRO are the ones that shape the WRL network. The producers and PRO will react to the instruments and make decisions that incentive their action (Forslind, 2005). Another example is a collection target that requires high participation from the consumers. The collection target will affect planning the location or reception points in more accessible locations such as supermarkets or arranging curbside collection.

Thus far, the performance of EPR is evaluated through the achievement of the targets set by the government. Separate targets for reuse, recycling and recovery can be linked to CE strategy in slowing and closing the loop.

Slowing the loop strategy on the product level is supported by design for durability as well as ease of repair and maintenance. Durability concerns the functionality of a physical product without the need for excessive maintenance. Material choice becomes important in ensuring that the products last longer. An example of durability in a product can be observed by the treadwear rating of tyres, where a higher rating indicates it lasts longer under normal operating conditions. Design for repair and maintenance allows the product to be maintained to sustain its function. These initiatives enable EEE, tyres and packaging to have a second life and stay longer in use, while design for disassembly and reassembly enables slowing the loop on the component level. It concerns the ease of separating different components of a product and reassembling it for reuse. Closing the loop is achieved through recycling, where the material is recovered without maintaining the physical geometry of the products. Design for recycling makes recycling feasible technically, environmentally and economically.

This literature overview demonstrates the importance of WRL and EPR. It is now being moulded together to show that WRL is the operational aspect of EPR. We present the architecture of policy instruments to identify what improvements can be made, and we describe WRL network design based on studies of the main activities of RL. Actors can use the variety of configurations from the WRL network to make decisions in arranging WRL.

## Methodology

This article uses a case study to identify how WRL in a collective EPR scheme in Finland contributes to CE. A case study can be considered as the object of research or methodology, which is defined as the ‘exploration of a bounded system or a case (or multiple cases) over time through detailed, in-depth data collection involving multiple sources of information rich in context’ (Creswell, 1998, p. 61). This article applied a case study as a methodology because it is suitable for examining contemporary events where the associated behaviour cannot be manipulated (Rowley, 2002; Yin, 2003). A case study can thoroughly describe the system being studied within its contextual conditions (Yin, 2003). This study uses a single case design, although multiple products were analysed separately. This type of study is called embedded-single-case, or a set of individual cases where multiple analysis is made to the subunits within one case to provide opportunities for comprehensive analysis and extensive insights (Gibbs, 2012; Yin, 2003). This case study explores and describes EPR in detail since the data or evidence are collected in various forms through different data collection tools. The sources included literature, public documents, email communications and interview. Therefore, the discrepancy between what is written in the literature and what is happening on a practical level with the PROs can be discovered. This method will also facilitate an investigation of important features and main problems within certain topics, providing insights into the direction of future studies.

This research follows the four steps of the case study strategy namely defining the aim, developing the instruments, gathering the data, analysing the data and disseminating it (Stuart et al., 2002).

Stage 1: Formulating the aim of the research. The research aimed to examine the WRL of the collective EPR system in Finland. It was translated into research questions as follows: (1) What is the system architecture of the policy instruments? and (2) How different or similar is the WRL network across different EoL products? These research questions showed the focus of the work was to investigate the process (the system architecture) and the outcome (how the RL scheme and its outcome under the EPR scheme are). The selection of six products subjected to EPR to build the case study was also conducted in this stage. Those six products are covered by the collective EPR scheme in Finland; therefore, the inclusion of all of them could provide comprehensive result on national level and enable comparison across different products. The products are batteries and accumulators, EEE, vehicles, packaging, tyres and paper. It applied a set of individual cases over time, resulting in a comparative historical study. Overall, 17 PROs are responsible for all the products. Furthermore, it is common in Finland to have a service company that organizes multiple PROs responsible for the same products (e.g. Rinki for PROs packaging or Elker for PROs WEEE). The study centred on the collective scheme since most of the products subject to the EPR in Finland are managed through a collective scheme (e.g. all producers of paper, tyre, ELV and beverage packaging join a collective scheme; Pirkanmaan ELY-keskus, 2020).

Stage 2: Developing the instrument. Document analysis complemented with personal communications were the selected instruments to conduct the case study. Firstly, an investigation was conducted on the implementation of EPR and RL through a literature study. The results were used as a basis to develop a strategy for data collection and analysis. The study on EPR implementation was a basis to assess the system architecture of the policy instrument. At the same time, literature studies on RL main activities would be utilized to analyse WRL network design. When developing the design framework for WRL, the main considerations were waste type and recovery options (Van Engeland et al., 2020). Hence, once the EoL products have been identified, the next step is identifying the journey from the point of the products being discarded. Meanwhile, personal communication with representatives of PRO and ELY-keskus, a government body that oversees the EPR scheme, was also conducted.

Stage 3: Gathering the data. Information on EPR practice was derived from publicly available data, including ones provided by the PROs, government authorities, logistics and recycling operators, etc. The data were complemented by personal communication to confirm certain issues through email exchanges with representatives of Serty (a consortium of PRO for EEE), Rescer (PRO for battery and accumulator), Suomen Autokierrätys (PRO for ELV), Suomen Kuitukierrätys Oy (PRO for fibre packaging) and Pirkanmaan ELY-keskus (government authority) as well as an interview with a representative from Rinki (a service company handling PRO for packaging). Email exchanges included but were not limited to requests for the latest data concerning EPR statistics, information on how the products were collected, who processed the waste (original manufacturer or operator) and where the location was, etc. A semi-structured arrangement was used for the interview to stimulate more discussion around the topic. The interviewer introduced the idea of the study around EPR and WM, followed by an opening question regarding the existing structure of EPR for packaging in Finland.

Stage 4: Analysing the data. The documents were analysed using a deductive approach where pointers were developed based on the research questions and initial literature to find necessary information. Patterns and themes based on the RQ were identified from the data (e.g. how the waste is collected, where the waste is reprocessed, any recycling target for specific products, financing type, etc.). Along the way, the inductive approach was incorporated when information deemed essential was found. The results of personal communications were evaluated, and some quotes were the opinion of the correspondents. The results of these stages were utilized to develop a design framework, identifying the level of product recovery and the circular strategies associated with each product.

The next step was determining the level of product recovery by examining whether the recovery was applied to the level of product, component or material. The circular strategy was then identified by inspecting the available recovery options (generated during design framework development) and examined using CE strategies namely slowing, closing and narrowing the loop (Bocken et al., 2016). They defined slowing the loop as prolonging and/or intensifying utilization of a product, closing the loop is achieved through recycling and narrowing the loop aims at utilizing less resource per product. These strategies were chosen because they are broad enough to cover the product life cycle, aligning with the CE position at the strategic level and can be used to define the circular approach in the product system and business model.

## Results

### The system architecture of the policy instruments

Administrative, economic and informative instruments were applied to all products. The summary of the system architecture of the policy instruments applied in the EPR scheme in Finland is shown in Table 2. Take-back and reporting obligations were found across all products (Pirkanmaan ELY-keskus, 2020, 2021). The former requires the producers to provide a separate collection scheme free of charge, and the latter obliges the producers to monitor the implementation and report the result to the Finnish authority. The responsible parties in the take-back arrangement are varied among all products. Although EPR aims to shift responsibility from municipalities to producers in managing waste, municipalities still have some role in EPR. Producers are the sole responsible party in take-back obligations for tyres, batteries, accumulators, paper and vehicles. In comparison, municipalities are involved in collecting waste packaging and EEE. Municipalities and individual PRO negotiate the contract on WEEE collection, whereas, for packaging, municipalities may handle some of the curbside collection.

**Table 2.** The system architecture of the policy instruments.

Instruments		EEE	Tyre	Packaging	Batteries and accumulators	Paper	Vehicles
Administrative instruments	Take-back obligation	Yes	Yes	Yes	Yes	Yes	Yes
	Collection target	45% of products in the past 3 years	95% of products placed on the market	–	45% of products in the past 3 years	–	–
	Treatment target	It is varied across EEE: • 55–80% reuse and recycling	All collected products should be recovered*	It is varied across different materials for about 16–80% recycling of products placed on the market	Recycling efficiency is varied about 50–75%	75% recycling of product placed on the market	The targets:• 85% reuse and recycling • 95 % recovery* of scrapped cars.
		• 75–85% recovery*					
Economic Instruments	Financial responsibility	Disposal fee	Disposal fee	Disposal fee	Disposal fee	—	Disposal fee
				Deposit/refund system (beverage)			
				Container tax (beverage)			
Informative instruments	Reporting obligation	Yes	Yes	Yes	Yes	Yes	Yes
	Labelling	Energy label					

EEE: electrical and electronic equipment.

* Recovery/recovered includes reuse, recycling and energy recovery.

Collection target and treatment target are other administrative instruments used to complement take-back obligations. Batteries and accumulators, EEE, tyres and packaging are regulated under the EU directive, requiring uniformity among member states for reporting purposes. The uniformity can be seen in how the EEE, batteries and accumulators collection targets were adopted from the EU directive. The estimation is based on the average quantity of products placed in the market within 3 years. For vehicles, there is no collection target to meet. Paper and packaging are the other products without a collection target. However, the treatment target of paper and packaging is based on the amount of product placed on the market, implying the minimum quantity to be collected. The treatment target refers to reuse, recycling and energy recovery. There is no stand-alone reuse target required by law across all products; instead, the target aims at reuse and recycling altogether.

The financial responsibility intends to cover collection, transportation, sorting and reprocessing. These activities are financed by the producers that integrate the cost of handling EoL products into the price of products. Differences concerning financial instruments are present across products. Paper is the only product that does not apply a disposal fee because the revenue could finance the scheme. The scheme’s effectiveness is affected by the market value of recycled paper in Finland (Deloitte, 2014). Other products are financed through a disposal fee estimated by the PRO and paid by the producers. An exception applies to beverage packaging, which combines the fee with a deposit–refund system and container tax.

The deposit–refund system is implemented to increase the collection rate of beverage packaging by paying back the deposit to consumers when the products are returned. The deposit–refund system is commonly used to manage EoL beverage packaging because these products cause litter and are difficult to regulate (Zhou et al., 2020). In Finland, this instrument is mixed with container tax. This tax requires producers to pay a levy for each container placed in the market. However, producers can get an exemption from paying container tax by joining a PRO. *This strategy will consolidate the return of beverage packaging through one channel to increase collection and recycling rate as well as take advantage of economies of scale* (PRO, packaging).

### WRL network design

The WRL was synthesized from the data collected during the study. Documents’ contents were analysed to find information regarding WRL logistics activities such as collection, sorting and reprocessing. Interview results and email communications complemented this analysis. Each activity was scrutinized deeper to gather information concerning collection arrangement and where the sorting and reprocessing occur. Furthermore, the recovery options implemented for each product were examined to assess the type of circular strategy being applied. Table 3 summarizes the RL of six cases concerning the collection method, inspection and recovery.

**Table 3.** Waste reverse logistics structure.

Products	Number of PRO	Collection				Inspection/sorting		Recovery	
		Curbside	Drop-off	Pick-up	Mobile	Centralized	Distributed	Original facility	Secondary facility
Accumulators and batteries	4		x			x			x
EE	5		x	x	x	x			x
Packaging	5	x	x			x	x	x	x
Beverage packaging	1		x				x		x
Paper	2	x	x				x	x	x
Tyres	1		x				x		x
Vehicles	1		x	x			x		x

PRO: producer responsibility organization; EE: electronic equipment.

Product recovery is about the location, whether it takes place in original or secondary facility, and the recovery options. The recovery can be performed on different levels: product levels, component levels and material levels through reuse, repair, remanufacture, repurpose, recycle and waste to energy (WtE) (Table 4). Although landfilling practice exists, it is not included in Table 4 since landfilling does not promote recovery at any product level.

**Table 4.** Waste recovery options.

Products	Product recovery options				
	Reuse/repair	Remanufacture	Repurpose	Recycle	WtE
Accumulators and batteries				x	x*
EE	x			x	x
Packaging	x			x	x*
Paper				x	x*
Tyres	x	x	x	x	x
Vehicles	x			x	x

EE: electronic equipment; WtE; waste to energy.

* The proportion is not explicitly mentioned in statistics, but some of these products end up in mixed waste and go into waste to energy plant.

**Figure 1.**
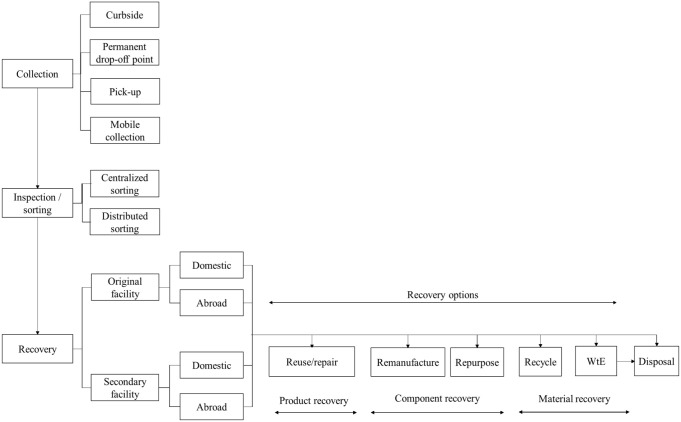
Waste reverse logistics framework under collective extended producer responsibility in Finland.

Figure 1 presents a framework for WRL developed based on WRL structure and recovery options within this study. The framework can be used to develop a new WRL network or change an existing scheme for either individual or collective schemes.

#### Collection

Consumers’ participation determines the collection rate, which defines the EPR’s success. The participation is affected by convenience, incentive and awareness. There are four collection schemes: curbside, drop-off, pick-up and mobile collection. *The collection of waste material is expensive, and when we have separate bins for several fractions, it is even more expensive. Our goal is to minimize the fees from customers (producers)* (PRO packaging).

The drop-off scheme is used in all six waste streams as it is required by law to organize nationwide reception points. The curbside method is applied for paper and packaging made of plastic, metal, glass and cardboard. Properties are responsible for arranging a separate collection bin except for housing in sparsely populated areas. The curbside scheme is particularly beneficial for low-value products with a short lifetime generated in high volume by households so that the scheme takes advantage of economies of scale. It was reported that the curbside collection would result in the highest collection rate for paper and packaging (Larsen et al., 2010). This scheme is convenient and encourages compliance.

For beverage packaging, the deposit–refund system incentivises the consumers to return the products to reception points instead of discarding them with other types of packaging. Zhou et al. (2020) reported a positive correlation between the deposit value and the return rate, although the ideal value was difficult to determine. This correlation was sensitive until the return rate was 90% (Lindhqvist, 2000). EEE, vehicles, batteries and accumulators, and tyres are collected mainly through the drop-off system. These products have a longer lifetime ranging from weeks to years, thus affecting the disposal frequency. The drop-off system is less costly than the curbside collection and relatively fast to implement. People are also willing to participate as long as the reception points are convenient (e.g. within 8 km for WEEE; Saphores et al., 2006; Wagner, 2013). Hence, the government regulate the number of reception points in Finland to reassure each population centre has access to it. Mobile collection for small WEEE is organized once or twice a year in sparsely populated areas to increase products’ return rates (Ylä-Mella, 2015). The last scheme is a pick-up, available for large WEEE and vehicles provided by a recycling operator or local environmental service authority. The success of the WEEE collection is attributed to the citizen’s awareness (Ylä-Mella et al., 2014). Furthermore, setting a separate scheme to recycle paper has been a tradition in Finland since the 1990s. Therefore, when the practice was expanded into other types of waste, it was not a foreign practice.

#### Inspection/sorting

Sorting can be conducted in a decentralized or centralized facility. Centralized facilities are common for products that require high-cost testing, specialized equipment and labour. Accumulators and batteries as well as WEEE are inspected in centralized systems because they require more complex inspection and trained labour. For packaging, plastic material is sorted in a centralized facility at which optical sorters based on infrared technology is used to distinguish different type of plastics for further treatment. On the other hand, decentralized sorting that occurs at the collection points is suitable for products with consistent and easily replicable testing (Barker and Zabinsky, 2008), as well as simple products whose conditions are commonly uniform when discarded (e.g. paper) and easily distinguished.

#### Recovery options

Among the six cases, part of the paper, cardboard and wooden packaging are reprocessed in the original facility, while other products are recovered in a secondary facility. The recovery can occur on the product, component or material levels which depends on the available technology, product characteristics and condition when discarded. Among the six products, EEE and tyres can undergo product, component and material recovery (for tyres, component recovery refers to retreading); packaging can be recovered on the product and material level; vehicles can be recovered on the component and material level, accumulators and batteries as well as paper can undergo material recovery.

From an economic perspective, material recovery (recycling) is not as expensive as collection, although the recycling costs vary across different materials. *Metal is the best; you get money when selling it. For other materials like household plastics, nobody wants to recycle them without the money in the system by the responsible companies* (PRO, packaging). It was also stated that policymakers saw EPR as a way to fund EoL management. People with first-hand experience managing EoL acknowledge how costly the system can be: *Waste is not valuable as they often read in the newspaper, ‘Someone’s waste is valuable to someone else,’ it is not when you think about money. It takes money to collect, sort and so on. You may get some money after that. We need somebody to pay, and now producers are more and more popular bodies to be responsible* (PRO, packaging).

### Collection and recovery performance

In general, the WRL across all products performs well compared to the target required by the legislation. Different collection strategies used in each product support the performance to achieve the collection target. Since the target came into force, the average collection rate for WEEE, tyres and batteries were about 51, 104 and 46%, respectively.

**Figure 2.**
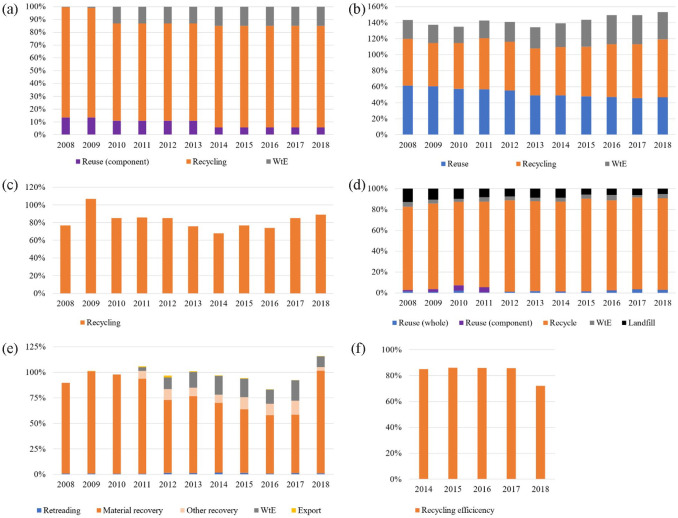
The recovery rate of (a) ELV, (b) packaging, (c) paper, (d) waste electrical and electronic equipment, (e) tyre and (f) batteries and accumulators.

Figure 2 shows the recovery performance of all products under the collective EPR scheme in the past 10 years, with recycling as the main contributor among other recovery options. The recovery options for ELV, packaging, paper, WEEE and tyre indicate the percentage of total products collected. However, for batteries and accumulators, the performance refers to efficiency, where it shows the ratio obtained by dividing the mass of recycling output by the mass of waste input. For packaging and EEE, the results were average values obtained from 5 and 10 different products, respectively. Reuse rate in packaging was comparable with the recycling rate, especially from 2008 to 2012, followed by a declining trend in reuse. For metal packaging, the reuse trend was consistent since its inherent properties allow multiple reuses. However, a major shift occurred in glass packaging, at which the reuse rate declined throughout the year. The reuse rate was around 45–65% from 2008 to 2011, then declined to 9 and 6%, respectively, in 2017 and 2018. The legislation change that did not require reuse for the refillable glass packaging caused the decline. The recycling of glass bottles was deemed to be more practical compared to reusing them.

In products such as packaging, paper and tyres, the total recovery rate reached more than 100%. It indicates that the waste is more than the product placed in the market. The treatment rate was calculated for tyres based on the product placed on the market in the corresponding year, although tyres can last up to a few years. A free rider is also a common issue within the EPR scheme, where the magnitude can be difficult to estimate. For packaging, the quantity of reused products was derived from previous years, whereas the denominator was based on products placed on the market in the corresponding year, causing a recovery rate of more than 100%.

### Circular economy strategies

Recovery options across different products are displayed in Table 4. These options, other than WtE, can be used to examine whether the products contribute to slowing or closing the loop. Although WtE allows a certain level of material recovery, this recovery option does not connect directly with product design. In other words, any design product can be processed in a WtE plant. Paper, portable accumulators and batteries are products that employ a closing-the-loop strategy through recycling. For batteries, promoting reuse started recently, with the primary target being batteries in electric vehicles (Pagliaro and Meneguzzo, 2019).

**Figure 3.**
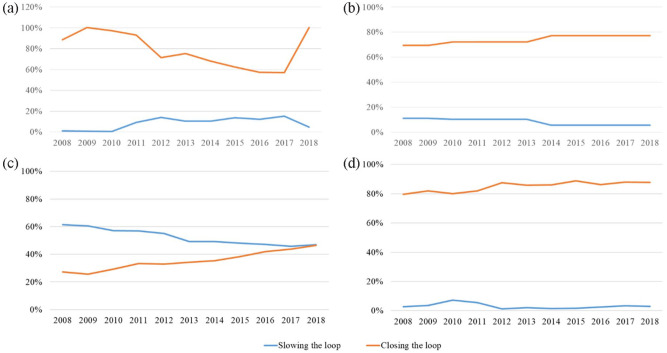
Recycling and reuse rate of (a) tyres, (b) ELV, (c) packaging and (d) waste electrical and electronic equipment.

Packaging, tyres, EEE and vehicles are products that close the loop and slow the loop through reuse/repair, remanufacture and repurpose. Figure 3 shows the circular strategies in the past decade for tyres, vehicles, packaging and EEE, where recycling and reuse can be categorized as closing and slowing the loop, respectively A similarity was found, where most of the products mainly rely on closing the loop through recycling. However, a different pattern was found in packaging, where the reuse trend decreased over the years while recycling increased. In order to compare the rate of reuse and recycling for packaging, the calculation was based on the sum of packaging placed in the market and the amount of reuse. One of the causes for the dynamic of reuse and recycling was a shift in the government regulation for beverage packaging. The decree on beverage packaging imposed a tax of 0.51 € per litre of beverage on producers who did not connect to the deposit–refund system. The tax made it expensive for producers to stay out of the deposit–refund system. The decree also required producers in the deposit–refund system to reuse their packaging. This law was then amended, and the reuse obligation was not required anymore. The recycling organization was simpler than reuse, causing the shift towards a lower level of product recovery.

## Discussions and limitations

### Main challenges: ELV and WEEE

Despite the well-performing EPR in Finland, some challenges are present and developments can improve EPR implementation. The future challenge in the EPR system in Finland is more pronounced in products with higher complexity such as vehicle and EEE. There was no collection target for ELV, and it has consistently had a collection rate of not more than 45% (Deloitte, 2014). Setting collection target for vehicles can be complex due to its long average age of 22 years, export practice and dismantling by unauthorized operators, and its active secondhand market (Autoalan Tiedotuskeskus, 2022; Inghels et al., 2016). Even though the handling may be environmentally sound, the dismantled car will not be reported as an unregistered vehicle. Expanding network operators can be a solution to this issue. More information is to notify the consumers regarding the legal way of disposing of vehicles and their authorized operators.

Legislation covering WEEE in Finland may not serve the main goal of EPR optimally. Among others, the legislation does not require a separate reuse target, and it is one of the most challenging issues within environmental policy (D’Amato et al., 2012; Horta Arduin et al., 2019). The reuse target has the potential to tap as the recast directive has suggested a 5% reuse target. A separate reuse target could promote higher recovery level, which aligns with the core of CE in resource management and value retention. However, there is a potential drawback of an increase in the overall environmental footprint (Esenduran et al., 2016). Older appliances consume high energy and may contain banned substances making them less attractive to reuse (Dalhammar et al., 2021).

Furthermore, the legislation necessitates EEE producers who serve private consumers to join PRO. This approach provides an advantage to the producers by removing their physical responsibility in handling EoL products and the economies of scale, leading to a more efficient network, especially for collection and transportation in more sparsely populated areas in Finland. Currently, collective scheme has become more favourable due to the general lifespan of EEE that has become shorter, hence no hassle for the original producers (Richter and Koppejan, 2016). As shown by smartphones, television, washing machine and vacuum cleaner, their average lifespan is 2.3 years shorter than the design or the desired lifetime (EEA, 2020). Joining PRO also means that the recovery will take place in a secondary facilities that will not interfere with the original manufacturing process; hence, the producer does not need to adjust the process in the original plant. A collective scheme through PRO will increase the reassurance of collection target fulfilment as the stream of similar products from different brands are taken care of altogether. The downside of processing in a secondary facility is that all products produced by various producers will be treated indiscriminately regardless the design or composition which not supporting higher value retention.

Another downside, a collective scheme does not support the notion of DfE. The idea of collecting same products produced by different producers and treated indiscriminately do not provide an incentive to improve its design. The shared obligations of a collective scheme can lead to a risk in equalizing cost and responsibility among various producers that can cause the lack of compliance on an individual level (Pouikli, 2020). Nonetheless, implementing proportional costs according to the actual brands’ WM costs can be difficult since it requires assessing treatment costs up to the product level. Atasu and Subramanian (2012) reported that charging a recovery fee based on the mix of waste generation does not always offer a favourable outcome. This method could result in low-end producers free-ride the high-end manufacturers. Hence, it was suggested that brand separation is applied when dealing with mixed WEEE. Nonetheless, solely from logistical perspective, collective scheme is seen as the most reasonable way: *This (collective scheme) is the only possibility and the best possibility for a single company to organize this (EPR). When they do it together through producer organization, that is the best way* (PRO, packaging).

### The importance of refining policy instruments and business model

Refining current policy instruments can improve EPR to achieve its main goals. Advanced fee modulation can be implemented to improve the DfE in a collective scheme. The European Commission endorses the member states to apply advanced fee modulation based on durability, reparability, reusability, recyclability and presence of hazardous substances (European Parliament, 2018). The conventional disposal fee, which is the form of basic fee modulation paid by the producers, reflects only the weight of the product placed on the market. Thus, the incentive is obtained by manufacturing a lighter product. The environmental aspect can be integrated into the system by applying advanced fee modulation. The producers need to provide a DfE factsheet of their products, including the lifespan of the products with normal usage. Some products, such as EEE, can take advantage of radio-frequency identification technology. It will provide information to the reader from the point of first selling until disposal. Therefore, the information from the DfE factsheet can be matched with the actual results of product recovery. Standardization will be needed on a European level to promote better recycling, covering a unified definition and terminology, uniform methodology in calculating the recycling rate, and defining the quality of secondary material. It can also support an international market in secondary materials by developing trust concerning the quality of secondary materials. Implementing either a penalty-only approach or modulated obligations is recommended to start advanced fee modulation practice, especially when multiple PROs exist (Ahlers et al., 2021). The first approach will require producers to continue paying a normal fee to the PRO, whereas producers with substandard products will pay the penalty. The penalty will be coordinated by an independent body to finance activities done by PROs related to WM. The latter would impose a bonus and penalty. PROs with many good producers will not only receive less contribution from the producers but also require to collect less waste. The opposite applies to PROs with substandard producers.

Another example is tyres, at which the average retreading rate in Finland was around 1% in the past 10 years (Finnish Tyre Recycling Ltd, 2022). The waste hierarchy will require higher-value recovery, making retreading a primary recovery option (e.g. Campbell-Johnston et al., 2020; Winternitz et al., 2019). However, this should be complemented by careful consideration of the type and quality of the products as well as whether higher-value recovery will compromise the products’ safety and functionality. Instead, more initiatives should support the aim of achieving DfE. By applying fee modulation and requiring a DfE factsheet, producers who can increase the durability will pay less. Moreover, when the products are used up to their lifespan and are not eligible for reuse, the recovery is still deemed a success although the higher-value recovery does not occur.

Tax and subsidy schemes can be introduced as an economic instrument in the EPR scheme in Finland. Introducing a tax and subsidy scheme can be seen as a means to promote DfE. The amount of tax companies pay depends on the product’s environmental performance. The collected tax is then given to the companies as a subsidy to support their innovation. The subsidy amount will be fixed regardless of the environmental performance of the companies. Therefore, the greenest producers will pay the least tax and gain the same subsidy. A model simulation done by Brouillat and Oltra (2012) reports that a tax and subsidy system can create radical innovation in the design for recycling. Combining a mandatory recovery target with tax and subsidy will stimulate innovation and improve the outcome compared to combining a mandatory target with an advanced disposal fee (Brouillat and Oltra, 2012; Dubois, 2012). The mandatory target in Finland can be revisited regularly and adjusted. Setting an EPR target is ideally based on the social, environmental and financial impacts.

The producers must also reconsider their business model, moving from selling to leasing products. This business model can promote the shift towards individual schemes and DfE. Leasing does not transfer product ownership from producers to consumers making producers responsible for the products throughout their life cycle. This type of arrangement gives producers individual responsibility for the products. Applying a leasing system can incentivize companies to manufacture long-lasting products because the products will be intensively used, cost and material efficient, and reused as much as possible at the EoL stage (Tukker, 2015). This proposition was confirmed by the economic model showing that compared with selling, leasing provides a higher incentive to produce durable products (Pangburn and Stavrulaki, 2014; Steeneck and Sarin, 2018). The policy should be designed accordingly to support the leasing scenario optimally. So far, mandatory targets have been used to evaluate the system’s performance. In the case of leasing, durability-dependent targets (a target tied to product failure) should be applied because mandatory targets may reduce the company’s willingness to manufacture durable products (Pangburn and Stavrulaki, 2014). Implementing a leasing model can start with producers who serve the business by using pilot projects targeting a few businesses where the relationship is already built.

### Transparency and economic implications

One of the aims of EPR, which was to shift the burden from the municipality, was reflected by the economic policy instruments found across different products. Disposal fee was universally used, except for paper, with additional policies such as a deposit–refund system and beverage tax for the beverage packaging. Among others, economic instruments combined with collection and recycling targets will affect the costs, benefits and how the WRL network is designed. The costs and benefits are born among actors involved in the network, which can be classified into collectors, sorters, recyclers and PROs. The same entity can have more than one role (e.g. recycler carries out sorting and recycling) or multiple entities covering the same role (e.g. WM company and company handle collection). PRO determines the value of the fee and distributes it to ensure that each actor within the WRL network bears an equal financial burden. However, uneven distributions can be found where the municipality that is commonly involved in the collection experiences financial losses. In the case of plastic packaging, the municipality was compensated by the PRO for about 60–70% of the total cost; hence, the deficit was covered using tax waste (Andreasi Bassi et al., 2020). For WEEE, the municipality was reimbursed for not more than 16% of its expense (Favot et al., 2016).

Among four actors within the WRL, recyclers are not connected directly to the PRO. They do not receive compensation from PRO as one of the financial benefits. Their revenues are from selling secondary materials or avoiding purchasing raw materials. Examples from plastics recycling showed the importance of the market substitution factor as the most sensitive parameter (Faraca et al., 2019; Mayanti & Helo, 2022). It implies market acceptance highly affects secondary material’s economic and environmental benefits. Hence, the economic viability of secondary material depends on the market demands, cost competition with virgin material and cooperation among actors within the WRL network (Andreasi Bassi et al., 2020). A continuous evaluation of the WRL is necessary to investigate the most suitable network configuration in order to obtain optimum outcomes. With regard to the methodology, this study did not particularly investigate the economic impacts of EPR scheme. Future studies can focus on the economic impact of EPR scheme by applying cost–benefit analysis covering capital expenditure (building and equipment costs) and operating expense (the cost of energy, material and wage maintenance) (Andreasi Bassi et al., 2020).

Within the context of the study, it demands more transparency for data regarding EPR practice in Finland to expand the study, including quantifying the economic impact. The required data are more than what has been reported, such as the quantities placed on the market, collected, recycled and utilized. Some PROs disclose the cost of the disposal fees; however, information concerning how and how much the municipality is being compensated is still unclear. The knowledge about costs and benefits distribution among actors can be utilized to improve the system since it provides information about the financial hotspot. Moreover, the economic feasibility of the system can be analysed continuously when the authority plans to revise the legislation (e.g. increasing collection or recycling target). The summary of challenges and opportunity regarding the EPR implementation can be found in Table 5.

**Table 5.** Summary of challenges and opportunity regarding extended producer responsibility implementation.

Item	Challenges	Opportunities/actions	Relevance with instruments
Improve ELV management	Unauthorize dismantler	Setting collection target	Administrative instrument
	Secondhand market	Expanding operator network	Informative instrument
		Information to the consumers regarding ELV disposal	
Improve WEEE management	Various condition of discarded WEEE	Setting reuse target (recast directive suggested 5% target)	Administrative instrument
	Legislation requiring B2C producers to join collective scheme	New reuse market	
Supporting DfE	The nature of collective scheme (e.g. same treatment to similar products from different producers)	Advanced fee modulation based on DfE requirements (e.g. durability, repairability, etc.)	Economic instrument
	Equalizing cost and responsibility among producers	DfE checklist sheet	Administrative instrument
	Low-end products can free ride high-end	Radio frequency identification (RFID) for trace and track	Informative instrument
		Leasing model	
		Applying durability-dependent targets	
		Tax and subsidy scheme	
Economic impacts	Potential uneven economic burden among actor	Fairer financial compensation from PRO	Economic instrument
		Improved data published by PRO	Informative instrument

DfE: design for the environment; PRO: producer responsibility organization; WEEE, waste electrical and electronic equipment.

### Methodology limitations and future works

Applying a case study methodology poses some strengths and limitations. Case studies can provide detailed accounts and insights on certain contemporary issues within their context. The variety of data used in a case study can offer insights and strengthen the study. For example, material recovery was seen as potential economic revenue to pursue in literature. In comparison, the interview with the PRO of packaging explicitly showed an account of how expensive the system is. It was highlighted that the system could be managed because producers put their money first, while the PRO continuously tries to reduce the fee. The case study can contextualize an issue, which in this case, provides an insight into the gap between the theory (waste is a resource) and the reality experienced by actors who are directly involved (waste is a waste). The other advantage of a case study is its design flexibility. A set of choices can be made to conduct a case study: the number of cases (single and several), variability (temporal and spatial), focus (process and outcomes), how to evaluate the data, etc. The flexible nature enables a case study to keep up with evolving research objectives since analysis in the case study begins while the data collection process is still ongoing, making the whole process iterative. However, the flexibility and comprehensiveness of a case study can cause drawbacks since a vast amount of data is needed, and they may not be available.

Other limitations of a case study are subjectivity and validity. The flexibility offers much freedom to build the study yet is prone to subjectivity. Decisions made during data collection, analysis and interpretation are influenced by researchers’ knowledge and what they consider important. Formulating research stages, as shown in Section ‘Methodology’, when working with a case study methodology can help with subjectivity. They provide guidelines such as the scope of the study, the required data, collection and analysis methods. Another issue with case study methodology is validity, where the study cannot be generalized statistically (as a sample representing a population). Nevertheless, it still provides insights since analytic generalization can be applied. The outcomes of a case study can result in a working hypothesis, lesson learned, principles deemed suitable for other situations and knowledge to support, alter or reject existing knowledge (Yin, 2003). This study supports what Barker and Zabinsky (2008) stated that decentralized or centralized sorting depends on the products and their sorting complexity. This study also verified their work, showing that most collective schemes use a secondary facility for reprocessing stage.

Future work can expand the study to other EU members to understand how their EPR system is. It will allow comparison and draw lessons in understanding the state of EPR implementation in different country members, primarily in understanding differences in policy instruments and policy design to achieve DfE as the main aim of EPR. Specific research about the effectiveness of financial instruments in reducing the waste or incentivizing DfE is also of interest. It can be conducted through an economic model to compare financial instruments such as disposal fees and deposit–refund systems for waste reduction.

## Conclusions

This article investigated the policy architectures and WRL networks of collective EPR in Finland for six different products: packaging, EEE, vehicle, batteries and accumulators, paper and tyres. Finland performs well in the collective EPR scheme, which can be associated to the system architectures of the policy instruments. It shows the significance of policy design and the stringency of the instruments. The positive outcomes associated with the EPR scheme are the results of a combination of different instrument as well as its stringency, since same instruments are implemented for different products but produce different outcomes. Nonetheless, DfE is still an issue to address. Refining existing policy instruments can assist the shift towards DfE. Careful consideration is necessary to formulate a policy that can support DfE such as DfE indicators, standardization, tax and subsidy systems and tariffs for disposal fees. Furthermore, the research highlighted the importance of policy to support circularity beyond closing the loop, which is still the main strategy within EPR. Finnish legislation that obliges EEE producers to join PRO will guarantee the fulfilment of recycling and energy recovery targets without prioritizing higher recovery value (e.g. product reuse or component reuse).

The study also generated WRL framework based on the existing EPR system in Finland. It facilitated assessment of the possible consequences due to differences in WRL network. The framework generated in this study can be used by PROs or producers to build WRL or switch from their existing system. The WRL networks were implemented to meet the target required by the administration instruments. Analysis of the WRL networks showed differences among Finnish PRO in managing the EoL products that was affected by waste type, quantity, value, cost, required sorting technique and available recovery options. The similarities in implementing a drop-off system and using secondary facilities were found across six products. This research further outlines the need for transparency. The publicly available information is insufficient to assess EPR performance comprehensively, particularly in quantifying the economic feasibility. Results showed multiple actors involved in the WRL networks that potentially experience uneven distribution of costs and benefits or even have a net loss. The interconnectedness among actors in the WRL networks also highlights their importance in linking the EoL stage with forward logistics to achieve circularity.

The research contributes to the limited number of current studies about EPR implementation which focusing on policy architecture and RL networks on a country level. The framework can serve as a guideline to develop efficient RL system while weighing the trade-offs. Moreover, the study puts EPR scheme into perspective since assessment on national level enables cross-comparison among products so that improvement on the network design or policy instruments combination can be improved by related actors.
